# Acidochromic Free-Standing Multilayered Chitosan-Pyranoflavylium/Alginate Membranes toward Food Smart Packaging Applications

**DOI:** 10.1021/acsapm.4c01085

**Published:** 2024-06-01

**Authors:** Mariana Cunha, Victor de Freitas, João Borges, João F. Mano, João M. M. Rodrigues, Luís Cruz

**Affiliations:** † REQUIMTE/LAQV, Department of Chemistry and Biochemistry, Faculty of Sciences, University of Porto, Rua do Campo Alegre, 687, 4169-007, Porto, Portugal; ‡ CICECO - Aveiro Institute of Materials, Department of Chemistry, University of Aveiro, Campus Universitário de Santiago, 3810-193 Aveiro, Portugal

**Keywords:** pyranoflavylium dye, biopolymers, layer-by-layer, pH-responsive biomaterials, click chemistry, food intelligent packaging

## Abstract

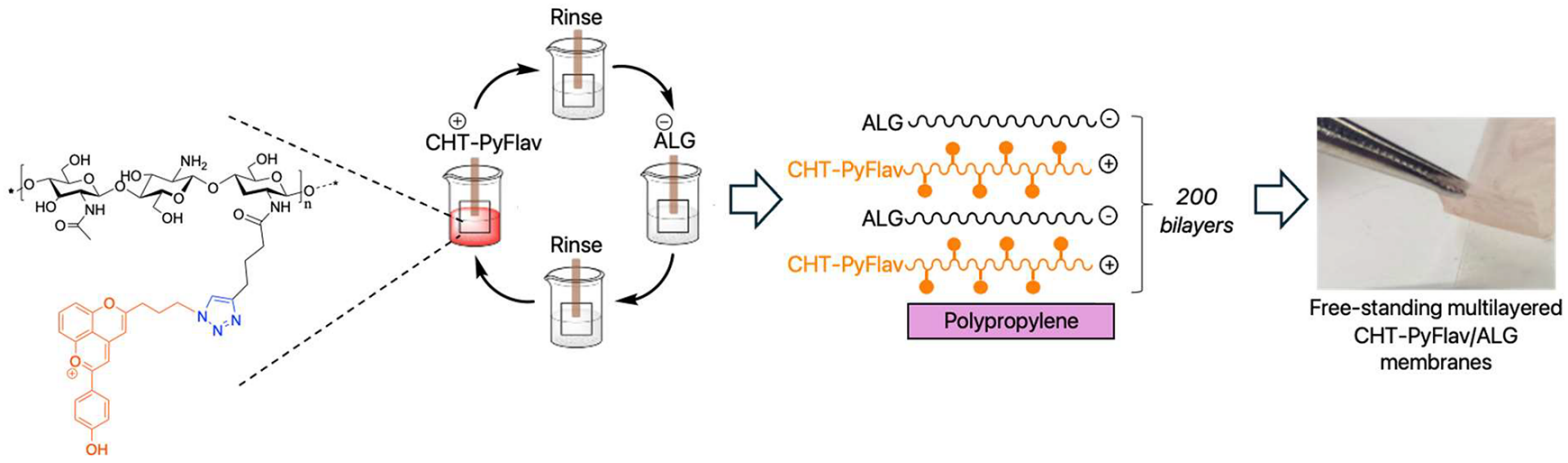

Food smart packaging has emerged as a promising technology to address consumer concerns regarding food conservation and food safety. In this context, we report the rational design of azide-containing pyranoflavylium-based pH-sensitive dye for subsequent click chemistry conjugation toward a chitosan-modified alkyne. The chitosan-pyranoflavylium conjugate was characterized by infrared (ATR-FTIR), ultraviolet–visible (UV–vis), nuclear magnetic resonance (NMR) spectroscopies, and dynamic light scattering (DLS), as well as its thermodynamic parameters related to their pH-dependent chromatic features. The fabrication of thin-films through electrostatic-driven layer-by-layer (LbL) assembly technology was first screened by quartz crystal microbalance with dissipation monitoring (QCM-D) onto gold substrates, and then free-standing (FS) multilayered membranes from polypropylene substrate were obtained using a homemade automatic dipping robot. The membranes’ characterization included morphology analysis and thickness evaluation, assessed by scanning electron microscopy (SEM), pH-responsive color change performance tests using buffer solutions at different pH levels, and biogenic amines-enriched model solutions, demonstrating the feasibility and effectiveness of the chitosan-pyranoflavylium/alginate biomembranes for food spoilage monitoring. This work provides insights toward the development of innovative pH-responsive smart biomaterials for advanced and sustainable technological packaging solutions, which could significantly contribute to ensuring food safety and quality, while reducing food waste.

## 1 Introduction

In the ongoing commitment to enhance food preservation and reduce food waste, the scientific community has shown increasing interest in food smart packaging as an approach to protect food from oxidation while providing information about the food shelf life on the package. As food spoils, proteins tend to break down into smaller substances such as volatile organic acids and nitrogen-based metabolites (e.g., biogenic amines), which can alter their pH values. For instance, during meat spoilage, the concentration of biogenic amines (BAs) tends to increase due to the amination and transamination of aldehydes and ketones during cellular metabolic processes, caused by the decarboxylation of amino acids to these compounds. As such, the ingestion of high concentrations of BAs (>50 mg·kg^–1^ in meat) can lead to symptoms such as nausea, diarrhea, headache, palpitations and high blood pressure, thus revealing low-quality and spoiled food presenting lack of hygiene standards.^1−4^

Having this in mind, several studies have been reported in the literature to assess the degree of conservation of perishable foods such as meat, fish, and dairy products using pH-sensitive natural dyes, namely anthocyanins, to develop colorimetric pH-responsive films that act as indicators of food freshness in real-time.^5−13^ However, the use of such dyes for these applications is still limited because they lose more than 50% of their color between pH 4 and 7, a critical range for monitoring color changes during food spoilage, due to the establishment of a complex chemical equilibrium network involving reversible reactions such as hydration, tautomerization, isomerization, as well as irreversible oxidative degradation mechanisms.^14−16^

In opposition, anthocyanin derivatives such as pyranoanthocyanins or pyranoflavylium-related dyes have recently emerged as promising alternatives to develop smart films for food packaging applications due to their superior chemical stability. The presence of an extra pyranic ring at the flavylium core, blocking the C-4 position, prevents the hydration reaction for the formation of colorless hemiketal species, thus avoiding tautomerization and isomerization reactions.^17,18^ Therefore, this type of dye undergoes only acid–base reactions, shifting from the yellow-orange acidic flavylium cation species to pink-blue neutral and anionic quinoidal base species.^19−21^ In a recent work, the design of one appropriate pyranoflavylium (Pyflav) dye and its noncovalent immobilization in an optimized cellulose acetate-based formulation was revealed to be crucial for achieving significant pH-dependent color variation without compromising stability.^22^ On the other hand, it was also demonstrated that Pyflav dyes could be chemically grafted onto marine-origin polysaccharides (alginate, chitosan) using carbodiimide coupling chemistry, offering a valuable strategy for developing pH-responsive bioconjugates.^23−25^ In this work, the well-known and efficient “click chemistry” was considered instead to covalently attach a pyranoflavylium-based dye onto a chitosan derivative.^26^ Therefore, an azide-containing Pyflav dye (PyFlav-N_3_) was first designed and synthesized and further functionalized to an alkyne-modified chitosan (CHT-Alk) via copper-catalyzed azide–alkyne cycloaddition reaction (CuAAC). Afterward, the fabrication of acidochromic free-standing (FS) membranes composed by alginate and chitosan-pyranoflavylium conjugate (CHT-PyFlav) was performed through bottom-up electrostatic-driven layer-by-layer (LbL) supramolecular assembly technology.^27,28^

In addition to producing functional membranes and thin-films with precise control over their thickness and composition, LbL assembly technology has proven to be an easy, affordable, and remarkably versatile technique for coating surfaces and creating a wide range of multifunctional layered structures with customizable compositions, architectures, properties, and functionalities. The deposition process can be influenced by different types of interactions, including electrostatic ones between charged polymers, hydrogen bonding, hydrophobic forces, and van der Waals forces. The main mechanism underlying LbL is primarily electrostatic interactions, which involve the sequential deposition of alternating layers of negatively and positively charged materials onto an inert substrate (e.g., polypropylene, PP). Several benefits are provided by this technology over casting-produced biomembranes, including the ability to control and regulate layer growth, presenting more uniform, highly reproducible membranes, and the possibility to incorporate rationally designed building blocks. Because membranes are produced in mild conditions, this technology is advantageous for use in sustainable chemistry and the food industry.^29−33^

The optimal LbL assembly conditions were first screened *in situ* onto gold substrates through quartz crystal microbalance with dissipation monitoring (QCM-D) technique and further, the production of FS multilayered membranes was attempted onto polypropylene substrates using a homemade automatic dipping robot.^32,34^ After morphological characterization by scanning electron microscopy (SEM), the colorimetric pH-responsive properties of the obtained membranes were validated by submersion in pH buffer solutions ranging from 4 to 7 and also when submitted to the headspace atmosphere of BAs model-like solutions.^35,36^

## 2 Experimental Section

### 2.1 Materials

Modified chitosan with hex-5-ynoyl groups (CHT-Alk), with a degree of substitution of 4%, a degree of acetylation of 4%, and a *M*_w_ = 150–250 kDa was kindly provided by Reykjavik University (Iceland). Alginic acid sodium salt (ALG) with α-L-guluronic acid (G) to β-D-mannuronic acid (M) (M/G) ratio of 1.56, *M*_w_ = 120–190 kDa was obtained from Sigma-Aldrich (Madrid, Spain). 5-Chloro-2-pentanone, sodium azide, copper(II) sulfate pentahydrate, sodium ascorbate, tris((1-benzyl-4-triazolyl)methyl)amine (TBTA), tetrabutylammonium iodide (TBAI), 2,6-dihydroxybenzaldehyde, 4-hydroxyacetophenone, putrescine (PUT), cadaverine (CAD), spermidine (SPD), tyramine (TYR), histamine (HIS) and ammonium hydroxide 25% (v/v) were purchased from Sigma-Aldrich. Dialysis cellulose membrane of 3.5 kDa molecular weight cutoff (MWCO) was purchased from Spectrum Laboratories, Inc. Theorell and Stenhagen universal buffer was obtained as described elsewhere.^37^

### 2.2 Synthesis of Chitosan-Pyranoflavylium Conjugate (CHT-Pyflav **6**)

#### 2.2.1 Synthesis of 10-Azidepropyl-4′-hydroxypyranoflavylium (PyFlav-N_3_**4**)

The synthesis of 10-azidepropyl-4′-hydroxypyranoflavylium **4** was prepared in two steps. First, 5-chloro-2-pentanone **1** (2 mL, 1 equiv) was added to a solution of sodium azide (3.4 g, 3 equiv) and TBAI (0.1% wt) in DMF (10 mL). The reaction was stirred overnight at 80 °C. After filtration of the remaining sodium azide and formed sodium chloride, ethyl acetate was added and washed three times with water. The organic phases were dried over anhydrous sodium sulfate, and the resulting mixture was filtrated. After solvent removal, the product 5-azido-2-pentanone **2** was obtained in oil form and used without purification (1.15 g, yield 50%). Then, 4′,5-dihydroxyflavylium dye **3** (50 mg, 1 equiv) was dissolved in EtOH:H_2_O 50:50 (v/v) solution (10 mL), and 5-azido-2-pentanone **2** (1.15 g, 50 equiv) was added and the pH set to 2.9. The reaction was stirred overnight at room temperature and monitored by HPLC-DAD. EtOH was evaporated, liquid–liquid extraction with ethyl acetate and water was carried out, and the aqueous phase was applied into a C18 silica gel column where the product 10-azidepropyl-4′-hydroxypyranoflavylium **4** was isolated with a solution MeOH:H_2_O 40:60 (acidified with HCl 2%). After MeOH evaporation, the fraction was freeze-dried where the pure product was obtained as an orange powder (3.39 mg, yield 10%). ^1^H NMR (600.13 MHz, DMSO-*d*_6_/TFA 90:10): δ (ppm) 8.24 (t, *J* = 8.9 Hz, H7), 8.22 (d, *J* = 8.9 Hz, H3′ and H5′), 7.91 (s, H3), 7.89 (d, *J* = 8.4 Hz, H8), 7.76 (d, *J* = 8.4 Hz, H6), 7.14 (s, H9), 7.07 (d, *J* = 8.9 Hz, H2′ and H6′), 3.51 (t, *J* = 6.6 Hz, H13), 3.00 (t, *J* = 7.4 Hz, H11), 3.00 (q, *J* = 6.8 Hz, H12). ^13^C NMR (125.77 MHz, DMSO-*d*_6_/TFA 85:15): δ (ppm): 172.13 (C3′ and C5′), 152.42 (C7), 120.40 (C8), 117.31 (C2′ and C6′), 113.55 (C6), 106.47 (C9), 102.88 (C3), 50.53 (C13), 32.33 (C11), 26.38 (C12). LC-DAD/ESI-MS [M]^+^*m*/*z* 346; [M-28]^+^*m*/*z* 318.

#### 2.2.2 Copper-Catalyzed Azide–Alkyne Cycloaddition (CuAAC)

For a 50 mL round-bottom flask, CHT-Alk **5** (100 mg, 0.55 mmol, 1 equiv) and Pyflav-N_3_**4** (1.5 mg, 0.004 mmol) were added and dissolved in DMSO (10 mL). A solution of CuSO_4_.5H_2_O (0.3 mM, 1 mL, 0.2 equiv) in water was added to an ice-cold solution of sodium ascorbate (0.6 mM, 1 mL, 0.4 equiv) and TBTA (1.5 mg, cat.) in water (2 mL). The resulting Cu(I) aqueous solution was transferred to the DMSO solution when a yellow precipitate appeared, yielding the final H_2_O:DMSO 1:2.5 (v/v) reaction mixture. The reaction was then allowed to stir at room temperature overnight and kept in the dark. The reaction mixture solution (yellow color) was transferred to a dialysis membrane with a molecular weight cutoff of 3.5 kDa. The dialysis bag was stirred in a beaker against deionized water for 1–2 days (water was changed twice every day). The solution was then taken out from the dialysis bag and lyophilized to get a final orange-red dried material. The chemical functionalization of CHT with Pyflav (CHT-Pyflav conjugate **6**) was assessed by proton nuclear magnetic resonance spectroscopy (^1^H NMR) and ATR-FTIR.

### 2.3 Determination of p*K*_a_ Value by UV–visible Spectroscopy

Spectrophotometric titrations of the free dye (Pyflav-N_3_) and conjugate (CHT-Pyflav) were performed to determine the respective p*K*_a_ values. First, a stock solution of free dye was prepared in EtOH:H_2_O 75:25 (v/v) solution with 0.1 M HCl (pH ∼ 1), whereas for the conjugate, a stock solution was prepared using a sodium acetate buffer solution at pH 3. In a quartz cuvette, different solutions were added in the following order: 1 mL of NaOH (0.1 M), 1 mL of universal buffer at desired pH, and 1 mL of the stock solution of the free dye, where the final solution was obtained with EtOH:H_2_O 25:75 (v/v) with the final concentration Pyflav-N_3_ (**4**) of 40 μg/mL. Titration experiments were performed with NaOH solutions (1 and 5 M) where increasing volumes were added to the cuvette, achieving different pH values, covering the pH range between 1 and 10. After each addition of base, the mixture was shaken, a UV–vis spectrum was recorded, and the final pH was measured. To obtain different pH values for the conjugate stock solution, 3 mL of the previously described stock solution (CHT-Pyflav (**6**) at 2.66 mg/mL) was transferred to a quartz cuvette, and the content was titrated with 1 and 5 M NaOH solutions. The p*K*_a_ values were taken from the least-squares method by using the Solver function of Microsoft Excel to fit the experimental values.

### 2.4 NMR Characterization

^1^H NMR (600.13 MHz) and ^13^C NMR (125.77 MHz) spectra were recorded using a Bruker-Avance 600 spectrometer working at 298.15 or 313.15 K, and TMS was used as an internal standard. The samples were prepared in DMSO-*d*_6_ with different amounts of acid or base (TFA). ^1^H chemical shifts were assigned using 2D NMR experiments (COSY), while ^13^C resonances were assigned using 2D NMR techniques (gHMBC and gHSQC). Multiplicities are expressed as singlet (s), doublet (d), triplet (t), chemical shifts (δ) in parts per million (ppm), and coupling constants (*J*) in hertz (Hz).

### 2.5 LC-DAD/ESI-MS

LC-DAD/ESI/MS analyses were carried out using the equipment and experimental conditions described elsewhere.^38^

### 2.6 ATR-FTIR

The FTIR characterization was performed in a PerkinElmer (model Spectrum Two), in the frequency range of 4000–400 cm^–1^, with 4 scans and a resolution of 4 cm^–1^.

### 2.7 Zeta-Potential Measurements

Stock solutions of native biopolymers and CHT-Pyflav **6** (1 mg/mL) were prepared in 0.1 M acetate buffer at pH 5.5 and diluted to a final concentration of 0.2 mg/mL for the evaluation of the net electrical charge of the individual solutions. The zeta (ζ)-potential values of the CHT-Pyflav **6** CHT-Alk **5** and ALG biopolymeric solutions were assessed at 25 °C using a Zetasizer Nano-ZS (Malvern Instruments Ltd., Royston, Hertfordshire, UK). An avalanche photodiode was used to measure the intensity of the light dispersed at a 173° angle, and a 633 nm laser was used to illuminate the solution. The data provided are an average of measurements performed in triplicates.

### 2.8 Quartz Crystal Microbalance with Dissipation Monitoring (QCM-D)

The electrostatic-driven LbL buildup of CHT-Pyflav/ALG multilayered films was monitored *in situ* by the fully automated QCM-D apparatus (Qsense, Biolin Scientific, Gothenburg, Sweden). Gold-coated 5 MHz AT-cut quartz sensors (QSX301 Gold, Q-sense, Gothenburg, Sweden) were used as substrates after being cleaned in a 1:1:5 (v/v) solution of NH_4_OH (25%), H_2_O_2_ (30%), and ultrapure water in an ultrasonic bath at 70 °C for 10 min, followed by rinsing with ultrapure water at room temperature and drying with a soft stream of nitrogen. The freshly cleaned Au-coated quartz sensors were inserted in the QCM-D chamber, equilibrated in an aqueous solution (0.1 M acetate buffer pH 5.5) until a stable baseline was achieved. Then, the Au-coated quartz crystal substrates were alternately exposed to 0.2 mg.mL^–1^ CHT-Pyflav (6 min adsorption time) and 0.2 mg.mL^–1^ ALG (6 min adsorption time) aqueous solutions in 0.1 M acetate buffer at pH 5.5. In-between the adsorption of the polycationic and polyanionic aqueous solutions, the substrate was rinsed with 0.1 M acetate buffer at pH 5.5 for 4 min to remove weakly adsorbed molecules. The assembly process was repeated five times until (CHT-Pyflav/ALG)_5_ multilayered thin films. In order to establish a stable baseline, the Au-coated quartz crystal was equilibrated with 0.1 M PBS pH 7.4 aqueous solution, the Au-coated quartz sensors were excited at several overtones (*n* = first, third, fifth, seventh, ninth, 11th, and 13th, corresponding to 5, 15, 25, 35, 45, 55, and 65 MHz, respectively), and the changes in the frequency (Δ*f*) and energy dissipation (Δ*D*) were monitored in real-time. The results shown in this work correspond to the frequency and energy dissipation shifts associated with the seventh overtone (*n* = 7; 35 MHz), respectively, after being normalized to the fundamental resonant frequency (5 MHz).^29,39^ The adsorption process was performed at 25 °C and a constant flow rate of 50 μL.min^–1^.

### 2.9 Fabrication of FS Multilayered Membranes

The buildup of biocompatible (CHT-Pyflav/ALG)_400_ FS multilayered membranes was attempted by repeating the sequential and alternate immersion of an inert, low-surface-energy polypropylene substrates into the oppositely charged biopolymeric aqueous solutions using a homemade automatic dipping robot (CORPUS, Guimarães, Portugal). Both the cationic CHT-Pyflav and anionic ALG biopolymeric solutions were prepared at 1 mg/mL in a 0.1 M acetate buffer at pH 5.5. In brief, the polypropylene substrate was alternatively and repetitively immersed in aqueous solutions of CHT-Pyflav (+) and ALG (−) for 6 min each until 400 CHT-Pyflav/ALG bilayers. In-between the adsorption of each oppositely charged polymer, the substrate was rinsed for 5 min in a 0.1 M acetate buffer aqueous solution at pH 5.5 to remove the weakly adsorbed layers and avoid the cross-contamination of the biopolymeric solutions. In the end of the assembly process, the FS multilayered membranes were dried in air prior to being easily detached from the underlying polypropylene substrate using just tweezers and kept in the fridge at 4 °C until use.

### 2.10 Scanning Electron Microscopy (SEM)

The morphology of CHT-Pyflav membranes was evaluated by using a high vacuum secondary electron mode field emission gun (FEG-SEM from Hitachi, model SU-3800, Tokyo, Japan) at working distances ranging from 4.7 to 5.4 nm and an acceleration voltage of 10 kV. The analytes were attached to a metal stub using double-sided carbon conductive tape to provide electrical contact. A Polaron E5000 sputter coater was then used to sputtered coat the analytes with a coating of conductive carbon (Quorum Technologies, Laughton, UK).^29^

### 2.11 pH-Sensing Properties in Food Spoilage Model-Like Solutions

The biocompatible free-standing membranes were split into 1.5 cm^2^ pieces and submerged in buffer solutions for 24 h. Buffer solutions of pH 4 and 5 were prepared using citric acid-phosphate buffer and pH 6, 7, and 8 buffer solutions were prepared using a sodium phosphate buffer. All pH measurements were performed in a Radiometer Copenhagen PHM240 pHmeter. The biomembranes were also positioned within a closed flask on a floating support above a 30 mL solution of either ammonium hydroxide or BAs. Cadaverine (CAD), putrescine (PUT), tyramine (TYR), histamine (HIS) and spermidine (SPD) were combined to create a BAs mixture or used separately with final concentration 4 g/L. Ammonium hydroxide solutions were performed at 0.35 and 3.5 g/L and stored overnight at 25 °C to mimic an amine-saturated environment as well. The color coordinates of the membranes were measured before and after immersion using a colorimeter, in triplicate.

Over time, the color difference was established through eq 1:
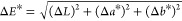
1where ΔL* = L* – *L*_0_*; Δa* = a* – *a*_0_*; Δb* = b* – *b*_0_*; *L*_0_*, *a*_0_*and *b*_0_* are values of the control membranes, which are the membranes before immersion.^40^

## 3 Results and Discussion

### 3.1 Synthesis and Structural Characterization

**Scheme 1:**
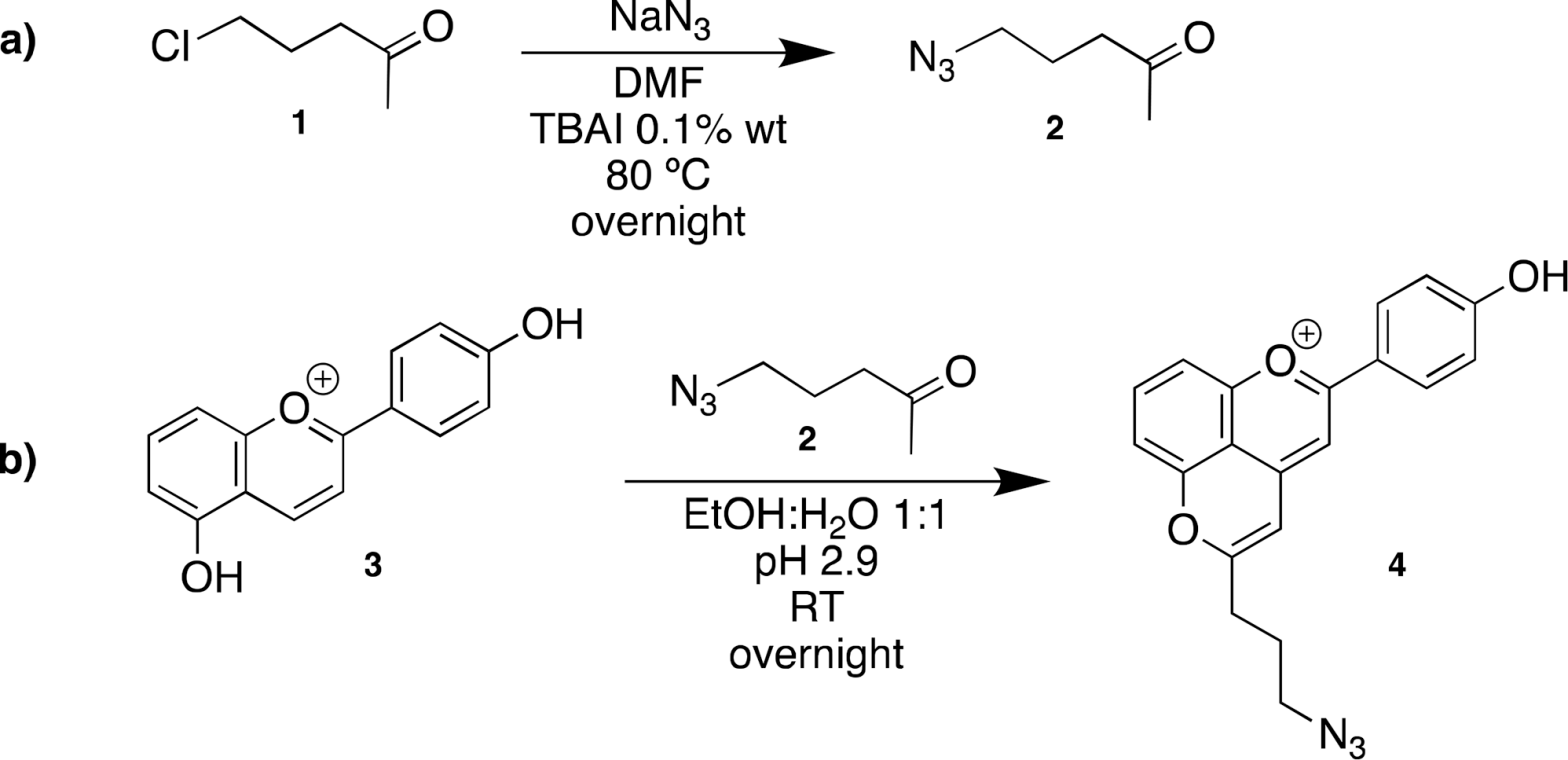
Schematic Representation of the Reaction Schemes to Obtain (a) 5-Azido-2-pentanone (**2**); (b) 10-Azidopropyl-4′-hydroxypyranoflavylium Dye (**4**) (Pyflav-N_3_)

The azidation of 5-chloro-2-pentanone (**1)** followed a classical SN_2_-type mechanism using sodium azide in DMF in the presence of catalytic amounts of Tetrabutylammonium iodide, TBAI (Scheme 1a). The reaction was stirred overnight at 80 °C and then filtered and treated with ethyl acetate. After solvent removal, 5-azido-2-pentanone **(2)** was afforded with 50% yield. The ATR-FTIR data confirmed the successful synthesis of compound **2** by the appearance of the typical band of the azide group around 2090 cm^–1^ (Figure S1, Supporting Information).

Further, following the well-established experimental conditions reported elsewhere, an annelation reaction between the previously obtained 4′,5-dihydroxyflavylium dye (**3**) and compound **2** was performed (Scheme 1b). The reaction followed the mechanism described for the formation of pyranoanthocyanins in acidic medium, namely, through the nucleophilic attack of compound **2** (in its enolic form) to the electropositive C-4 of flavylium cation. Afterward, the 5-OH adds to the carbonyl group, and after H_2_O elimination and oxidation steps the pyrano-derivative **4** was afforded.

The reaction progression was monitored by HPLC-DAD and after mixture treatment and column chromatography purification, the pure compound **4** was characterized by LC-DAD/ESI-MS in positive ion mode and ATR-FTIR (Figures S2–S3, Supporting Information). The molecular ion at [M]^+^*m*/*z* 346 agrees with the pyranoflavylium cation structure as well as the MS^2^ fragment, [M-28]^+^*m*/*z* 318 corresponding to loss of an N_2_ residue, a typical fragmentation found for the azide group. It was possible to observe the typical band of the azide group around 2090 cm^–1^ as well as the stretching bands of OH groups between 3500 and 3000 cm^–1^. The pigment was then structurally characterized by ^1^H NMR spectroscopy as well as by 2D NMR techniques (COSY, HSQC, HBMC) where all protons and carbons were assigned to the corresponding structure (Figure S4–S7, Supporting Information).

**Scheme 2:**
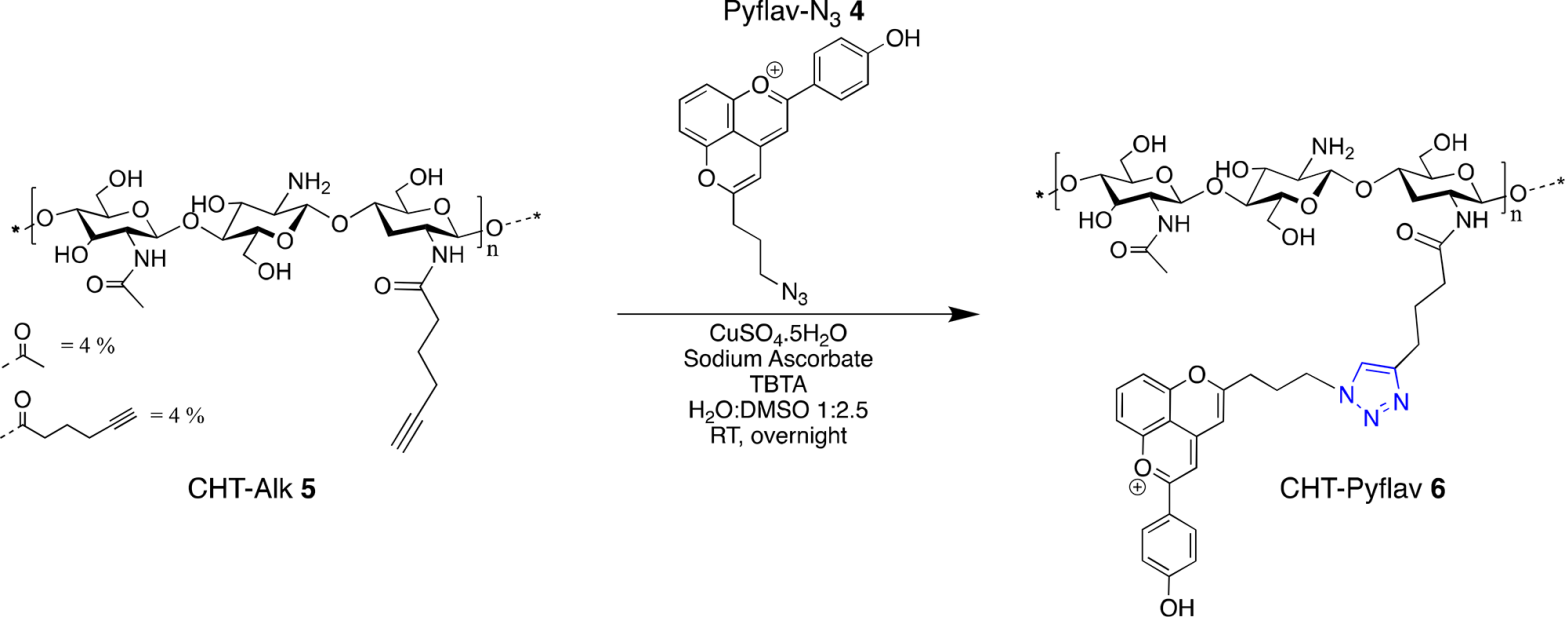
Schematic Illustration of the Reaction to Obtain CHT-Pyflav Conjugate **6** through Copper-Catalyzed Azide–Alkyne Cycloaddition Reaction (CuAAC)

Regarding the covalent conjugation of the dye (Pyflav-N_3_**4**) to the chitosan-modified alkyne (CHT-Alk **5**), a copper-catalyzed azide–alkyne cycloaddition reaction (CuAAC) was carried out (Scheme 2).

**Figure 1:**
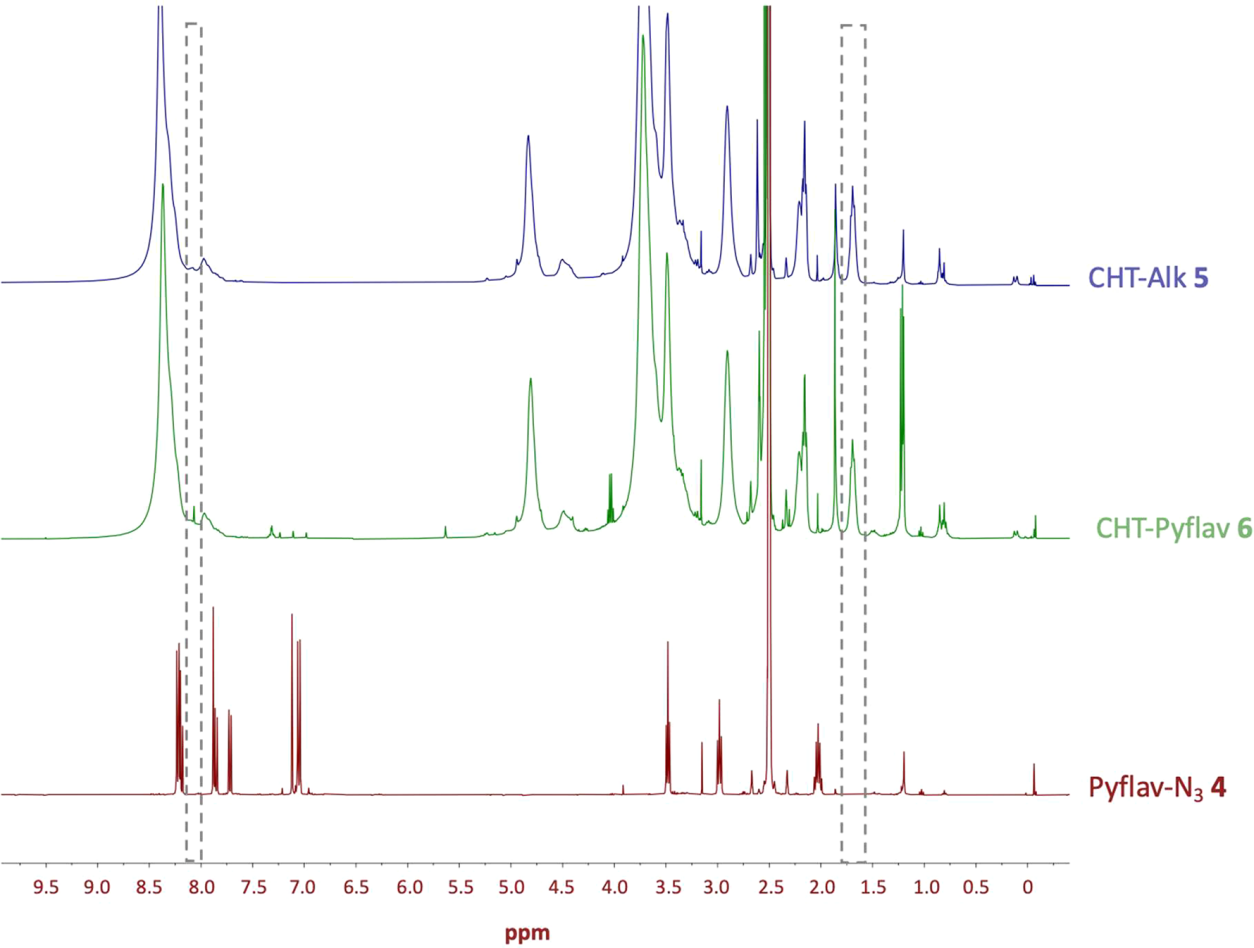
Proton nuclear magnetic resonance (^1^H NMR) spectra of Pyflav-N_3_**4**, CHT-Alk **5**, and CHT-Pyflav **6** recorded in DMSO-*d*_6_/TFA (85:15).

**Figure 2:**
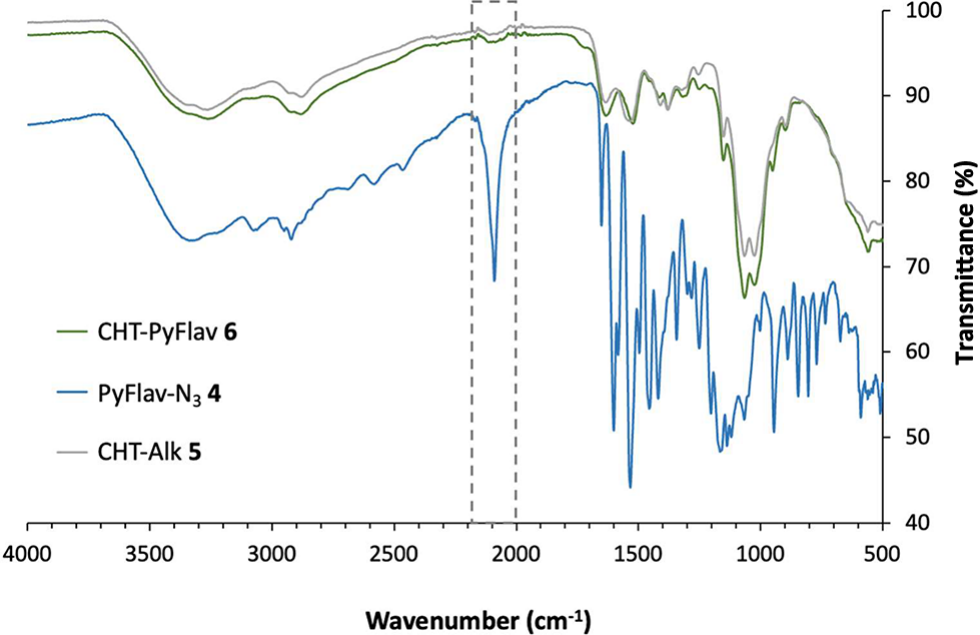
ATR-FTIR spectra of CHT-Alk **5**, Pyflav-N_3_**4**, and CHT-Pyflav **6**.

The reaction was stirred at room temperature overnight and then transferred into a dialysis membrane in order to remove unreacted Pyflav-N_3_**4** as well as all other reagents. After freeze-drying, the sample was characterized by ^1^H NMR, and ATR-FTIR (Figures 1–2).

Through the ^1^H NMR spectra (Figure 1) of the CHT-Pyflav conjugate, it was possible to confirm the appearance of signals in the aromatic region (7–7.3 ppm) which are assigned to the protons present in the pyranoflavylium dye. Moreover, the triazole group, which results from the conjugation between CHT-Alk and Pyflav-N_3_, can be assigned to the singlet at 8.0 ppm, which agrees with what is described in the literature (between 7 and 10 ppm).^41^

Additionally, it can be witnessed a decrease in the peak corresponding to the alkyne group, at 1.7 ppm,^42^ in the CHT-Pyflav spectrum comparing with the one of CHT-Alk.

In Figure 2, The disappearance of the azide group band in the spectra of the CHT-Pyflav conjugate can be well observed, which is a good indication of the success of the click chemistry reaction and the consequent formation of the triazole ring.

### 3.2 Physicochemical Characterization

The net electrical charge of the freshly prepared 0.2 mg.mL^–1^ ALG and CHT-Pyflav aqueous solutions in 0.1 M acetate buffer at pH 5.5 were found to be −24.5 ± 0.4 mV and +21.8 ± 0.5 mV, respectively, revealing the anionic and cationic nature of ALG and CHT-Pyflav biopolymers (Figure S8). Thus, we hypothesized that the LbL alternate deposition of oppositely charged ALG and CHT-Pyflav biopolymeric materials could enable the development of electrostatically driven multilayered thin films and FS multilayered membranes.

**Figure 3:**
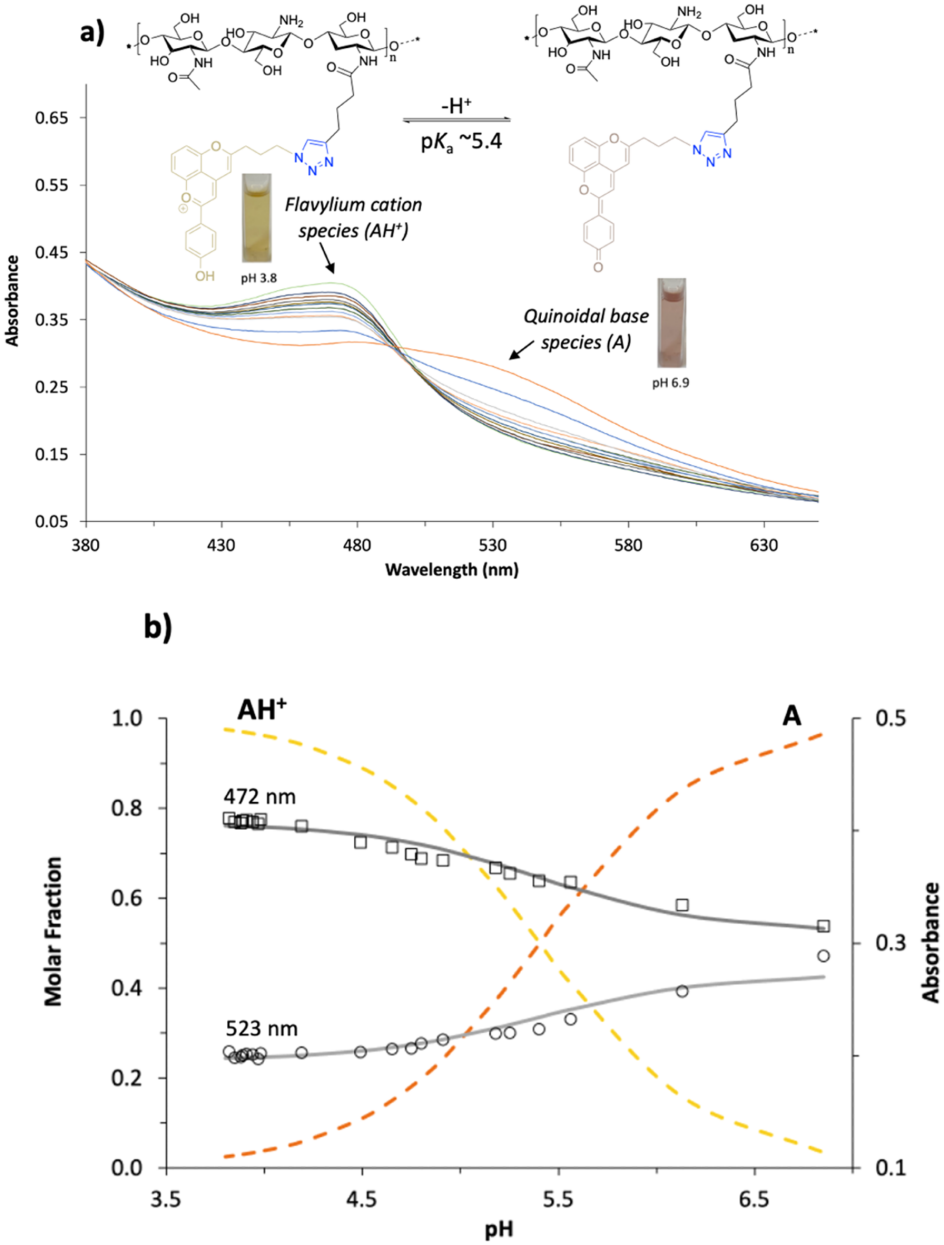
a) Spectral variations of CHT-Pyflav **6** as a function of pH and color displayed by the equilibrium species in acetate buffer. b) Molar fraction distribution of AH^+^ and A chemical species and fitting of the experimental data at two fixed wavelengths in function of pH to achieve p*K*_a_ = 5.4 ± 0.1.

To get the characterization of the acidic thermodynamic constant (*K*_a_) of the Pyflav-N_3_**4** (Figure S9, Supporting Information) and CHT-Pyflav **6** acid–base spectrophotometric titrations were performed (Figure 3).

The equilibrium between the cationic pyranoflavylium species (AH^+^) and the quinoidal base (A) is described by the following equation:
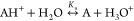
2
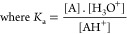
3

Taking into account that the sum of individual species concentration is *C*_0_ = [AH^+^] + [A], their mole fraction distribution (χ_*i*_) was calculated by the simplified equations: 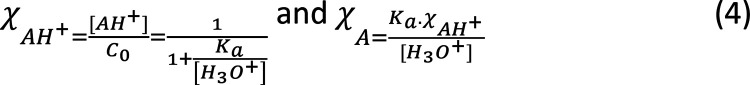
4

The fitting of experimental data at a given wavelength (λ) was by the following equation:
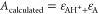
5where ε_AH+_ and ε_A_ represent the mole absorption coefficients of individual species.^18^

The absorbance values were calculated at different wavelengths and then fitted to the respective experimental data using the solver function to minimize the sum of the least-squares method. The determined p*K*_a_ values for the free dye and respective conjugate were 6.6 ± 0.5 and 5.4 ± 0.1, respectively, attributed to the deprotonation reaction of the hydroxyl group at C-4′ of the pyranoflavylium cation to give the quinoidal base species. The relatively lower p*K*_a_ value obtained for the conjugated derivative might be explained by the acidic character of chitosan, which may tune a higher stabilization for the quinoidal base.

### 3.3 Buildup of the Multilayered Thin Films and Fabrication of Free-Standing Biomembranes

The LbL assembly of CHT-Pyflav/ALG multilayered thin films was evaluated *in situ* by the QCM-D technique by applying an alternate field across the gold-plated quartz crystal substrate.

**Figure 4:**
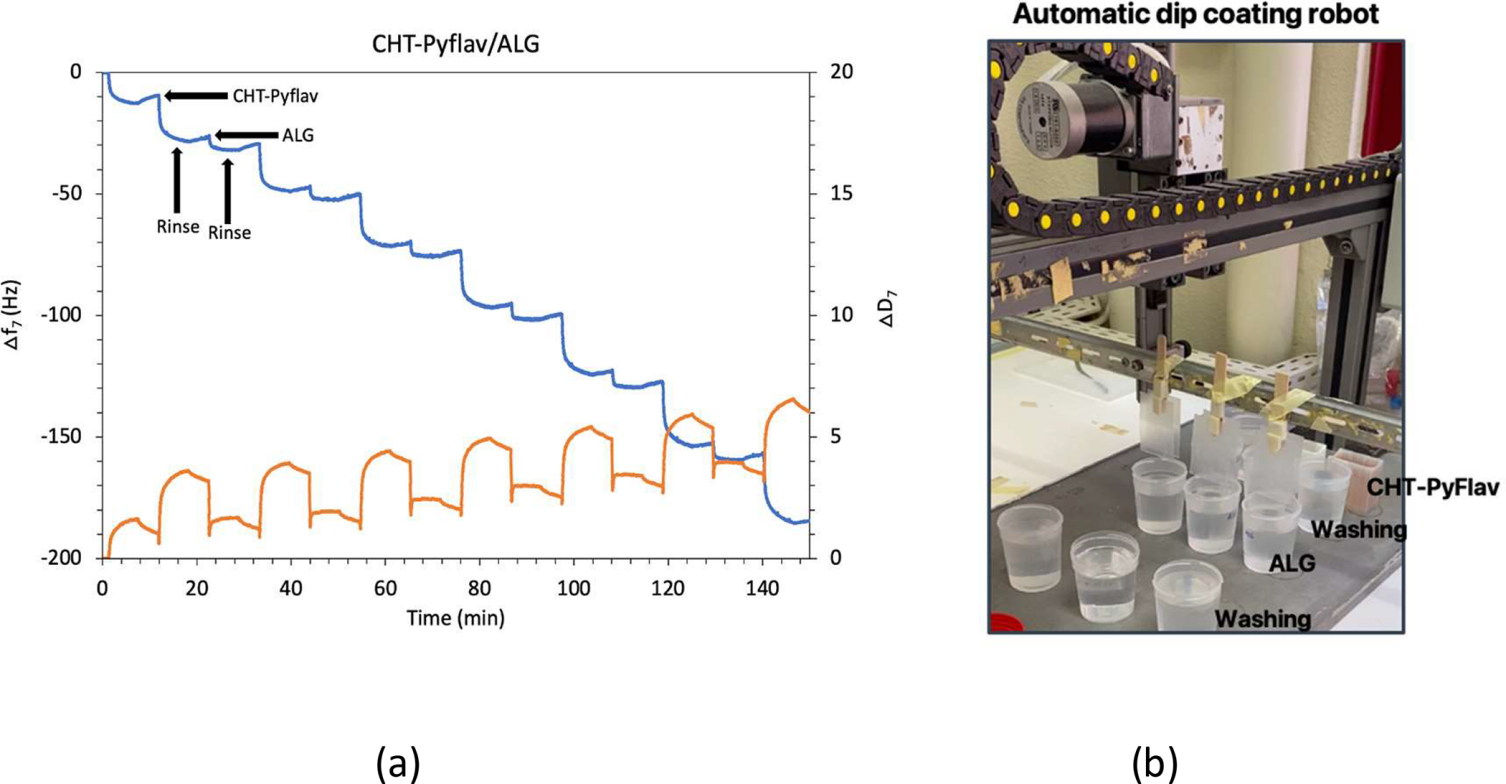
(a) QCM-D monitoring of the normalized frequency (Δ*f*_7_*/7*) and dissipation (Δ*D*_7_) shifts at the 7th overtone as a function of time for the buildup of (CHT-Pyflav/ALG)_7_ multilayered thin films in 0.1 M acetate buffer at pH 5.5 onto the Au-plated quartz crystal sensors. The inset numbers refer to the adsorption of (1) CHT-Pyflav and (3) ALG and (2,4) rinsing steps; (b) Fabrication of FS (CHT-Pyflav/ALG)_400_ multilayered membranes onto polypropylene substrates using an automatic homemade dip coating robot.

The QCM-D is more than a mass sensing device, allowing us to measure minute changes in mass per cm^2^, based on the changes in the frequency shift (Δ*f*_*n*_/*n*), but also infer the viscoelastic properties (ΔD_*n*_) of the adsorbed layer via the energy dissipated in the mechanical oscillation of the crystal. The obtained results in Figure 7 showed how the multilayered thin membranes develop after each successive material has been adsorbed. As shown in Figure 4a, the sequential decrease in Δf_7_/7 over time after the deposition of each biopolymeric solution indicates the increase in the hydrodynamic mass per adsorbed layer and the successful buildup of CHT-Plyflav/ALG multilayered thin films. Moreover, the increase in ΔD_7_ reveals the viscoelastic nature of the adsorbed layers, which is a typical behavior showcased by soft polymeric films. However, the higher increase in both Δf_7_/7 and ΔD_7_ after the deposition of ALG, reveals the stiffer nature of CHT-Pyflav layers in comparison with ALG.^27,29,32^

Thicker and robust self-standing multilayered membranes encompassing (CHT-Pyflav/ALG)_400_ layers were produced on an automatic dipping robot by repeating the alternate immersion of polypropylene substrates into 1 mg.mL^–1^ oppositely charged aqueous solutions of CHT-Pyflav and ALG in 0.1 M acetate buffer at pH 5.5, followed by air drying and easy detachment (Figure 4b).

### 3.4 Characterization of Multilayered Biomembranes

#### 3.4.1 Scanning Electron Microscopy (SEM)

**Figure 5:**
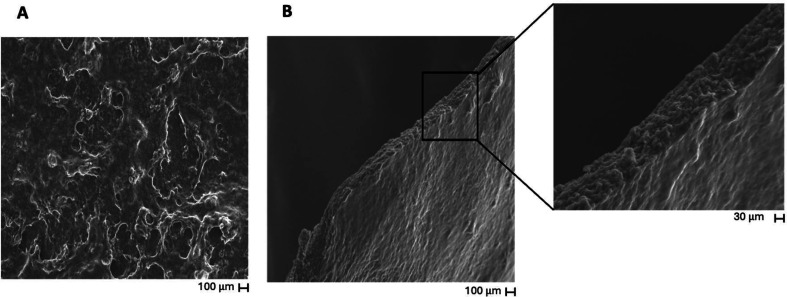
SEM micrographs of (CHT-Pyflav/ALG)_400_ FS multilayered membranes produced in 0.1 M acetate buffer at pH 5.5 onto polypropylene substrates. The scale bars represent 100 μm (A and B) and 30 μm (zoomed-in image of B).

The morphology and thickness of the biocompatible (CHT-Pyflav/ALG)_400_ FS membranes were evaluated by SEM. The SEM analysis revealed that the FS membranes exhibit a smooth homogeneous surface, denoting some degree of porosity, with a thickness of 11.9 ± 0.3 μm (Figure 5). It was also feasible to measure their thickness using a digital coating thickness meter (model TE 1250–0.1 F, Sauter), revealing a thickness of 15.4 ± 0.9 μm.

#### 3.4.2 Tests as pH-Indicators in Buffer Solutions and Biogenic Amines Solutions

**Figure 6:**
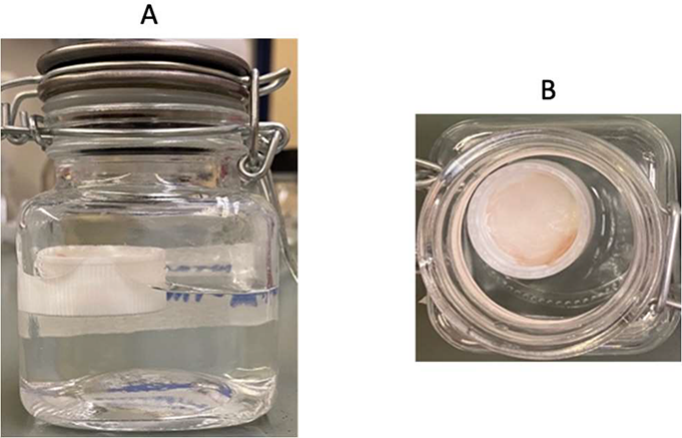
Photographs of the methodology used to expose the FS membranes to different solutions. Herein the FS membranes were placed in the headspace of closed flasks, front view (A) and top view (B), containing the buffer/BAs solutions.

The FS membranes were submitted to a pH-dependent colorimetric variation assay by immersing them in buffer solutions with pH values ranging from 4 to 7, which represents the critical pH interval of food spoilage. For the BAs solutions (4 g·L^–1^), the membranes were put into the headspace (Figure 6A and B) to simulate an amine-rich environment that occurs during the food deterioration process.

First, the biomembranes were put in contact with the buffer solutions at different pH values (4–7) for 24 h.^40^ After that time, the different membranes were analyzed by a colorimeter, obtaining the results presented in Figure S10 and Table S1, in Supporting Information.

Taking into consideration that p*K*_a_ of the conjugate is around 5.4 and the polyelectrolyte solutions used in the LbL assembly were set to pH 5.5, it was expected to achieve pale pink colored biomembranes. When they came into contact with more acidic solutions (pH of 4, 5, and even 6), their color clearly changed from pink to yellow. At pH 7, the membranes start to revert to their pale pink original color.

**Figure 7:**
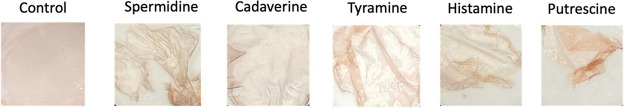
Images of small portions of the assembled membranes obtained after 72 h of exposure to BA solutions (4 g/L).

After that, the biomembranes were also submitted to colorimeter tests where they were put previously in contact with several BAs, whose results are shown in Table 1 and Figure 7, where it is also noticeable the color changing. Additionally, the ΔE* parameters are shown, illustrating the difference between the control color of the membranes and the various pH values to which it is exposed. The greater the ΔE* values, the less the observed color difference between the biomembranes; conversely, the greater the ΔE*, the greater the color difference and, thus, the greater the reactivity to pH and BAs.

**Table 1:**
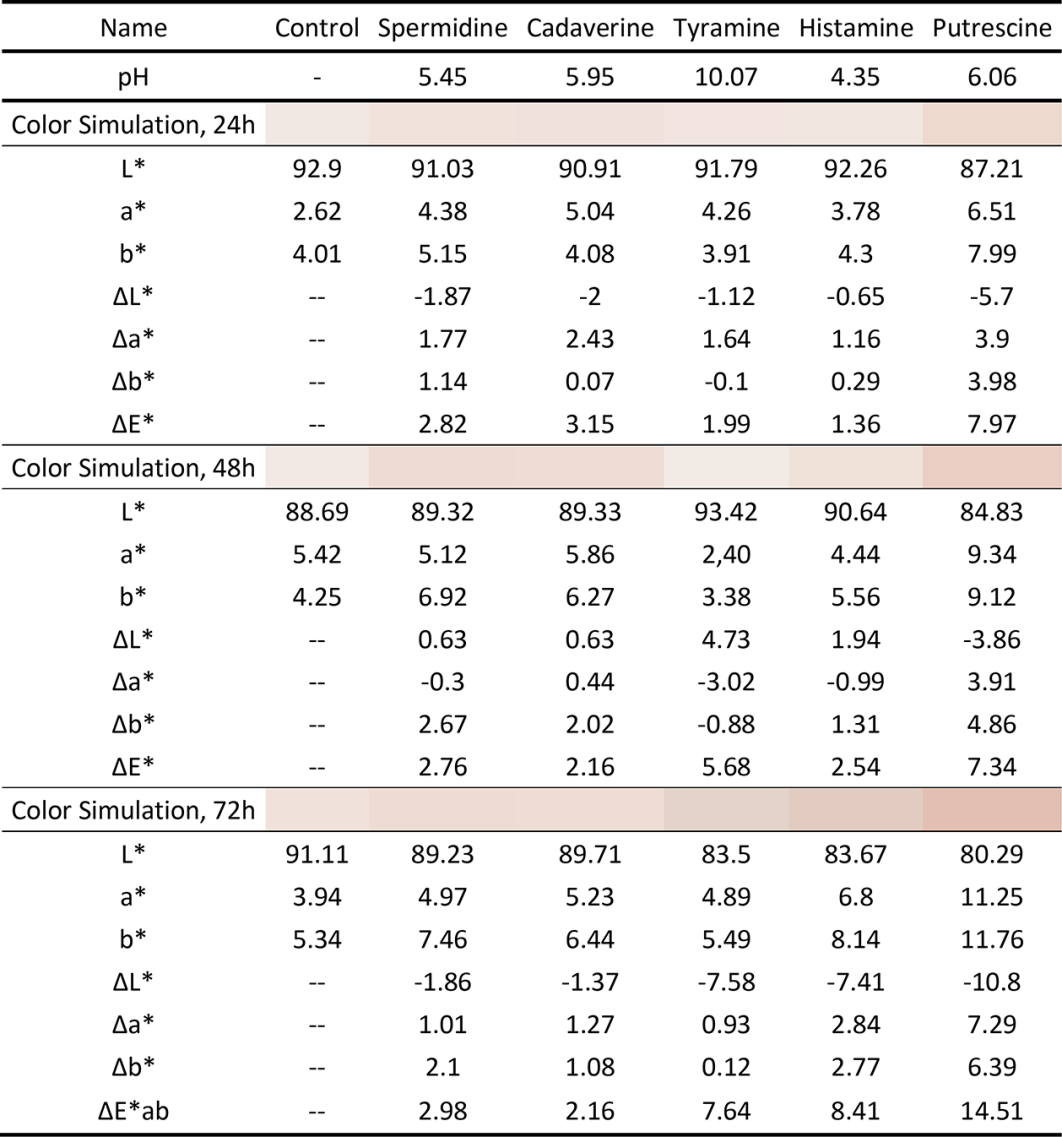
Colorimetric Data from the Membranes Taken at 24, 48, and 72 h after Immersion in Distinct BAs Solutions (4 g/L)

The colorimeter results corroborate the results harvested at the naked eye: in more acidic environments, the membranes turn yellow, and as pH values increase, the pink color of the membranes intensifies.

As it can be observed, the BA that had the greatest effect on changing the color of the membranes, and which also obtained a higher ΔE*, was putrescine (followed by histamine and tyramine), which may indicate that these biomembranes would be more suitable and sensitive for detection of this kind of biomarker during the food spoilage process. Despite their inherent fragility, efforts are underway to strengthen them due to their great color-changing potential.

## 4 Conclusions

In this work, the click chemistry reaction was demonstrated to be an efficient methodology for the covalent functionalization of chitosan-modified alkyne with rationally designed 10-azidepropyl-4′-hydroxypyranoflavylium dye, whose synthesis and characterization were also fully presented. The CHT-Pyflav conjugate was then used to fabricate FS (CHT-Pyflav/ALG)_400_ multilayered membranes through powerful LbL bottom-up technology. As shown throughout this work, the membranes exhibited great chromatic variation toward buffer solutions within the pH range of 4–7 and the sensing ability of BAs-rich environments, which both constitute typical conditions during food spoilage. A more pronounced chromatic change was noticeable in solutions with pH values lower than 5, where the membranes appear more yellowish, where the Pyflav dye presents itself in the cation AH^+^ form. On the other hand, when the membranes were submitted to higher pH environmental levels (pH ∼ 6–7), they started to become darker pink colored, thus revealing a potential indicator of food deterioration. Regarding BAs sensing response at the same concentration, the membranes were revealed to be much more sensitive (greater ΔE*) to putrescine detection rather than the other BAs.

Overall, the results demonstrate the viability of using natural polysaccharides and pigments bioinspired by natural anthocyanins to create smart and functional biomaterials that greatly interest food packaging applications.

## Supporting Information Available

HPLC-DAD/ESI-MS, ATR-FTIR, 1D and 2D NMR spectra (^1^H, COSY, HSQC, HMBC), and acidic constant parameter determination for the PyFlav dye; Chromatic variation of biomembranes in buffer solutions at different pH values, after exposure to ammonium hydroxide and biogenic amines solutions (PDF)

## References

[ref1] de la Torre C. A. L.; Conte-Junior C. A. Detection of Biogenic Amines: Quality and Toxicity Indicators in Food of Animal Origin. Food Control and Biosecurity 2018, 16, 225–257. 10.1016/B978-0-12-811445-2.00006-4.

[ref2] Feddern V.; Mazzuco H.; Fonseca F. N.; De Lima G. J. M. M. A Review on Biogenic Amines in Food and Feed: Toxicological Aspects, Impact on Health and Control Measures. Animal Production Science 2019, 59, 608–618. 10.1071/AN18076.

[ref3] Schirone M.; Esposito L.; D’onofrio F.; Visciano P.; Martuscelli M.; Mastrocola D.; Paparella A. Biogenic Amines in Meat and Meat Products: A Review of the Science and Future Perspectives. Foods 2022, 11, 788 10.3390/foods11060788. 35327210 PMC8947279

[ref4] Hernández-Jover T.; Izquierdo-Pulido M.; Teresa Veciana-Nogués M.; Mariné-Font A.; Carmen Vidal-Carou M. Biogenic Amine and Polyamine Contents in Meat and Meat Products. J. Agric. Food Chem. 1997, 45, 2098–2102. 10.1021/jf960790p.

[ref5] Zhang J.; Zou X.; Zhai X.; Huang X. W.; Jiang C.; Holmes M. Preparation of an Intelligent PH Film Based on Biodegradable Polymers and Roselle Anthocyanins for Monitoring Pork Freshness. Food Chem. 2019, 272, 306–312. 10.1016/j.foodchem.2018.08.041. 30309548

[ref6] Mao L.; Wang C.; Yao J.; Lin Y.; Liao X.; Lu J. Design and Fabrication of Anthocyanin Functionalized Layered Clay/Poly(Vinyl Alcohol) Coatings on Poly(Lactic Acid) Film for Active Food Packaging. Food Packag Shelf Life 2023, 35, 101007 10.1016/j.fpsl.2022.101007.

[ref7] Chen L.; Wang W.; Wang W.; Zhang J. Effect of Anthocyanins on Colorimetric Indicator Film Properties. Coatings 2023, 13, 1682 10.3390/coatings13101682.

[ref8] Zhu B.; Zhong Y.; Wang D.; Deng Y. Active and Intelligent Biodegradable Packaging Based on Anthocyanins for Preserving and Monitoring Protein-Rich Foods. Foods 2023, 12, 4491 10.3390/foods12244491. 38137296 PMC10742553

[ref9] Kaushani K. G.; Rathnasinghe N. L.; Katuwawila N.; Jayasinghe R. A.; Nilmini A. H. L. R.; Priyadarshana G. Trends in Smart Packaging Technologies for Sustainable Monitoring of Food Quality and Safety. International Journal of Research and Innovation in Applied Science 2022, 07 (07), 07–30. 10.51584/IJRIAS.2022.7702.

[ref10] Ardila-Diaz L. D.; Oliveira T. V. d.; Soares N. d. F. F. Development and Evaluation of the Chromatic Behavior of an Intelligent Packaging Material Based on Cellulose Acetate Incorporated with Polydiacetylene for an Efficient Packaging. Biosensors (Basel) 2020, 10 (6), 59 10.3390/bios10060059. 32486501 PMC7345045

[ref11] Wang Q.; Chen W.; Zhu W.; McClements D. J.; Liu X.; Liu F. A Review of Multilayer and Composite Films and Coatings for Active Biodegradable Packaging. npj Science of Food 2022, 6, 18 10.1038/s41538-022-00132-8. 35277514 PMC8917176

[ref12] Sobhan A.; Muthukumarappan K.; Wei L. A Biopolymer-Based PH Indicator Film for Visually Monitoring Beef and Fish Spoilage. Food Biosci 2022, 46, 101523 10.1016/j.fbio.2021.101523.

[ref13] Rodrigues C.; Souza V. G. L.; Coelhoso I.; Fernando A. L. Bio-based Sensors for Smart Food Packaging—Current Applications and Future Trends. Sensors 2021, 21, 2148 10.3390/s21062148. 33803914 PMC8003241

[ref14] Castañeda-Ovando A.; Pacheco-Hernández M. de L.; Páez-Hernández M. E.; Rodríguez J. A.; Galán-Vidal C. A. Chemical Studies of Anthocyanins: A Review. Food Chem. 2009, 113, 859–871. 10.1016/j.foodchem.2008.09.001.

[ref15] Pina F.; Melo M. J.; Laia C. A. T.; Parola A. J.; Lima J. C. Chemistry and Applications of Flavylium Compounds: A Handful of Colours. Chem. Soc. Rev. 2012, 41 (2), 869–908. 10.1039/C1CS15126F. 21842035

[ref16] Cruz L.; Basílio N.; Mateus N.; De Freitas V.; Pina F. Natural and Synthetic Flavylium-Based Dyes: The Chemistry behind the Color. Chemical Reviews 2022, 122, 1416–1481. 10.1021/acs.chemrev.1c00399. 34843220

[ref17] Rentzsch M.; Schwarz M.; Winterhalter P. Pyranoanthocyanins - an Overview on Structures, Occurrence, and Pathways of Formation. Trends Food Sci. Technol. 2007, 18 (10), 526–534. 10.1016/j.tifs.2007.04.014.

[ref18] Sousa J. L. C.; Gomes V.; Mateus N.; Pina F.; de Freitas V.; Cruz L. Synthesis and Equilibrium Multistate of New Pyrano-3-Deoxyanthocyanin-Type Pigments in Aqueous Solutions. Tetrahedron 2017, 73 (41), 6021–6030. 10.1016/j.tet.2017.08.051.

[ref19] Cruz L.; Petrov V.; Teixeira N.; Mateus N.; Pina F.; Freitas V. De. Establishment of the Chemical Equilibria of Different Types of Pyranoanthocyanins in Aqueous Solutions: Evidence for the Formation of Aggregation in Pyranomalvidin-3- O -Coumaroylglucoside-(+)-Catechin. J. Phys. Chem. B 2010, 114 (41), 13232–13240. 10.1021/jp1045673. 20879715

[ref20] Pina F.; Roque A.; Melo M. J.; Maestri M.; Belladelli L.; Balzani V. Multistate/Multifunctional Molecular-Level Systems: Light and PH Switching between the Various Forms of a Synthetic Flavylium Salt. Chem.—Eur. J. 1998, 4 (7), 1184–1191. 10.1002/(SICI)1521-3765(19980710)4:7<1184::AID-CHEM1184>3.0.CO;2-6.

[ref21] Pagan A.; Lee J. I.; Kang J. Concentration-Dependent Association of Flavylium Chloride with Differential Hydroxy Moieties in Ethanol. Colorants 2022, 1 (1), 20–37. 10.3390/colorants1010004.

[ref22] Gomes V.; Bermudez R.; Mateus N.; Guedes A.; Lorenzo J. M.; de Freitas V.; Cruz L. FoodSmarTag: An Innovative Dynamic Labeling System Based on Pyranoflavylium-Based Colorimetric Films for Real-Time Monitoring of Food Freshness. Food Hydrocoll 2023, 143, 108914 10.1016/j.foodhyd.2023.108914.

[ref23] Pires A. S.; Gomes V.; Neves D.; Mateus N.; De Freitas V.; Cruz L. Colorimetric PH-Responsive Biomaterials Based on Pyranoflavylium-Biopolymer Hybrid Conjugates. ACS Appl. Polym. Mater. 2022, 4 (7), 4961–4971. 10.1021/acsapm.2c00514.

[ref24] Cammarata C. R.; Hughes M. E.; Ofner C. M. Carbodiimide Induced Cross-Linking, Ligand Addition, and Degradation in Gelatin. Mol. Pharmaceutics 2015, 12 (3), 783–793. 10.1021/mp5006118. PMC5585870 25658665

[ref25] Wickramathilaka M. P.; Tao B. Y. Characterization of Covalent Crosslinking Strategies for Synthesizing DNA-Based Bioconjugates. J. Biol. Eng. 2019, 13, 63 10.1186/s13036-019-0191-2. 31333759 PMC6621941

[ref26] Li H.; Aneja R.; Chaiken I. Click Chemistry in Peptide-Based Drug Design. Molecules 2013, 18, 9797–9817. 10.3390/molecules18089797. 23959192 PMC4155329

[ref27] Sousa C. F. V.; Monteiro L. P. G.; Rodrigues J. M. M.; Borges J.; Mano J. F. Marine-Origin Polysaccharides-Based Free-Standing Multilayered Membranes as Sustainable Nanoreservoirs for Controlled Drug Delivery. J. Mater. Chem. B 2023, 11 (28), 6671–6684. 10.1039/D3TB00796K. 37377032

[ref28] Jeon H.; Noh J.; Jo M.; Joo C.; Jo J.; Lee C. Layer-by-Layer Engineered Flexible Functional Film Fabrication with Spreadability Control in Roll-to-Roll Manufacturing. Polymers (Basel) 2023, 15 (11), 2478 10.3390/polym15112478. 37299278 PMC10255543

[ref29] Monteiro L. P. G.; Borges J.; Rodrigues J. M. M.; Mano J. F. Unveiling the Assembly of Neutral Marine Polysaccharides into Electrostatic-Driven Layer-by-Layer Bioassemblies by Chemical Functionalization. Mar Drugs 2023, 21 (2), 92 10.3390/md21020092. 36827133 PMC9964173

[ref30] Qu B.; Luo Y. A Review on the Preparation and Characterization of Chitosan-Clay Nanocomposite Films and Coatings for Food Packaging Applications. Carbohydrate Polymer Technologies and Applications 2021, 2, 100102 10.1016/j.carpta.2021.100102.

[ref31] Cai M.; Zhang X.; Zhong H.; Li C.; Shi C.; Cui H.; Lin L. Ethyl Cellulose/Gelatin-Carboxymethyl Chitosan Bilayer Films Doped with Euryale Ferox Seed Shell Polyphenol for Cooked Meat Preservation. Int. J. Biol. Macromol. 2024, 256, 128286 10.1016/j.ijbiomac.2023.128286. 38000577

[ref32] Borges J.; Mano J. F. Molecular Interactions Driving the Layer-by-Layer Assembly of Multilayers. Chemical Reviews 2014, 114, 8883–8942. 10.1021/cr400531v. 25138984

[ref33] Criado-Gonzalez M.; Mijangos C.; Hernández R. Polyelectrolyte Multilayer Films Based on Natural Polymers: From Fundamentals to Bio-Applications. Polymers 2021, 13, 2254 10.3390/polym13142254. 34301010 PMC8309355

[ref34] Yan Y.; Björnmalm M.; Caruso F. Assembly of Layer-by-Layer Particles and Their Interactions with Biological Systems. Chem. Mater. 2014, 26, 452–460. 10.1021/cm402126n.

[ref35] Danchuk A. I.; Komova N. S.; Mobarez S. N.; Sergey; Doronin Y.; Burmistrova N. A.; Markin A. V; Duerkop A. Optical Sensors for Determination of Biogenic Amines in Food. Anal. Bioanal. Chem. 2020, 412, 4023–4036. 10.1007/s00216-020-02675-9/Published. 32382967 PMC7320057

[ref36] Omer A. K.; Mohammed R. R.; Mohammed Ameen P. S.; Abas Z. A.; Ekici K. Presence of Biogenic Amines in Food and Their Public Health Implications: A Review. Journal of Food Protection 2021, 84, 1539–1548. 10.4315/JFP-21-047. 34375430

[ref37] Küster F. W.; Thiel A. Tabelle per Le Analisi Chimiche e Chimico-Fisiche, 12th ed.; Ulrico Hoepli Editore, 1982.

[ref38] Cruz L.; Basílio N.; Mateus N.; Pina F.; De Freitas V. Characterization of Kinetic and Thermodynamic Parameters of Cyanidin-3-Glucoside Methyl and Glucuronyl Metabolite Conjugates. J. Phys. Chem. A 2015, 119 (5), 2010–2018. 10.1021/jp511537e. 25622073

[ref39] Marx K. A. Quartz Crystal Microbalance: A Useful Tool for Studying Thin Polymer Films and Complex Biomolecular Systems at the Solution - Surface Interface. Biomacromolecules 2003, 4, 1099–1120. 10.1021/bm020116i. 12959572

[ref40] Gomes V.; Pires A. S.; Mateus N.; de Freitas V.; Cruz L. Pyranoflavylium-Cellulose Acetate Films and the Glycerol Effect towards the Development of PH-Freshness Smart Label for Food Packaging. Food Hydrocoll 2022, 127, 107501 10.1016/j.foodhyd.2022.107501.

[ref41] Mungalpara D.; Kelm H.; Valkonen A.; Rissanen K.; Keller S.; Kubik S. Oxoanion Binding to a Cyclic Pseudopeptide Containing 1,4-Disubstituted 1,2,3-Triazole Moieties. Org. Biomol Chem. 2017, 15 (1), 102–113. 10.1039/C6OB02172G. 27805227

[ref42] Liu Z.; Liao Q.; Yang D.; Gao Y.; Luo X.; Lei Z.; Li H. Well-Defined Poly(N-Isopropylacrylamide) with a Bifunctional End-Group: Synthesis, Characterization, and Thermoresponsive Properties. Des. Monomers Polym. 2013, 16 (5), 465–474. 10.1080/15685551.2012.747165.

