# Supplementary material for: Acidochromic Free-Standing Multilayered Chitosan-Pyranoflavylium/Alginate Membranes toward Food Smart Packaging Applications
Source: ACS Appl Polym Mater. 2024 Jun 1;6(11):6820–30. doi: 10.1021/acsapm.4c01085 (PMC11186008; doi:10.1021/acsapm.4c01085)
Supplement: Supplementary file 1 — ap4c01085_si_001.pdf [file ap4c01085_si_001.pdf]

Supporting Information

Acidochromic free-standing multilayered chitosan-pyranoflavylium/alginate membranes towards food smart packaging applications

Mariana Cunha[a], Victor de Freitas[a], João Borges[b], João F. Mano[b], João M. M. Rodrigues[b,*], Luís Cruz[a,*]

[a]REQUIMTE/LAQV, Department of Chemistry and Biochemistry, Faculty of Sciences, University of Porto, Rua do Campo Alegre, 687, 4169-007, Porto, Portugal.
[b]Department of Chemistry, CICECO—Aveiro Institute of Materials, University of Aveiro, 3810-193 Aveiro, Portugal.

*Corresponding authors: Luís Cruz - luis.cruz@fc.up.pt; João M. M. Rodrigues - jrodrigues@ua.pt

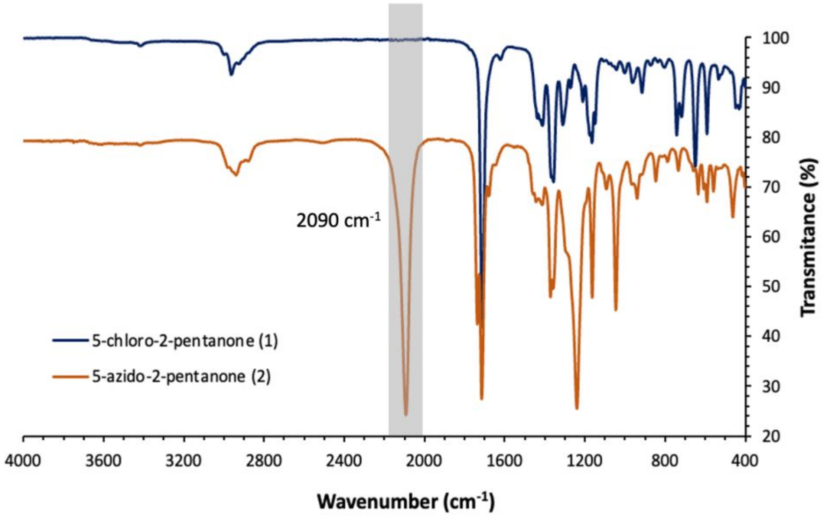

Figure S1. ATR-FTIR spectra of compounds **1** and **2**.

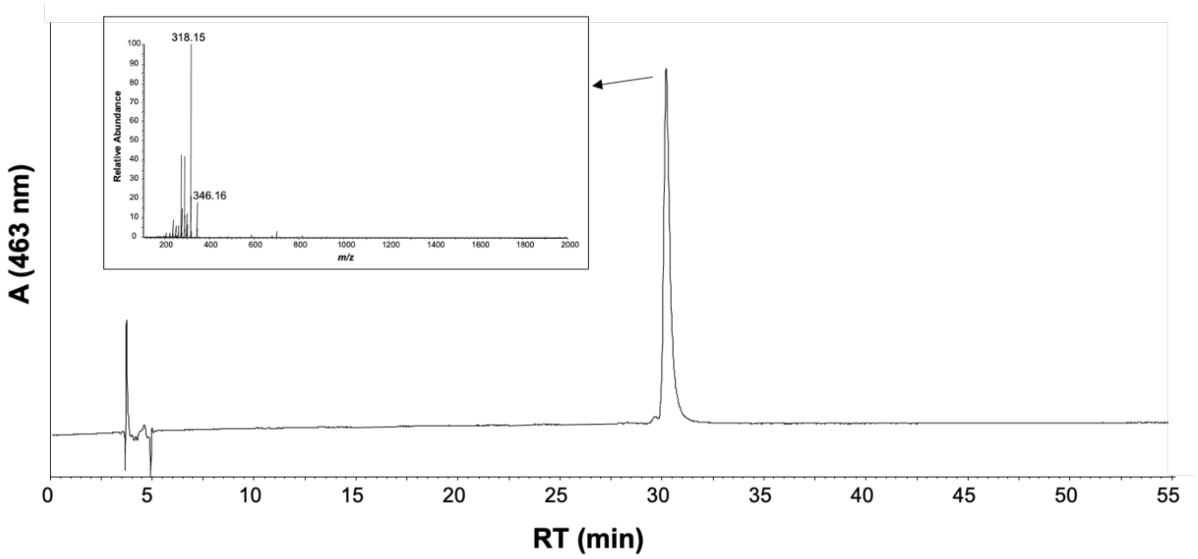

Figure S2. HPLC chromatogram of Pyflav (**4**) at maximum absorbance wavelength. Inset: Full MS spectrum of Pyflav (**4**).

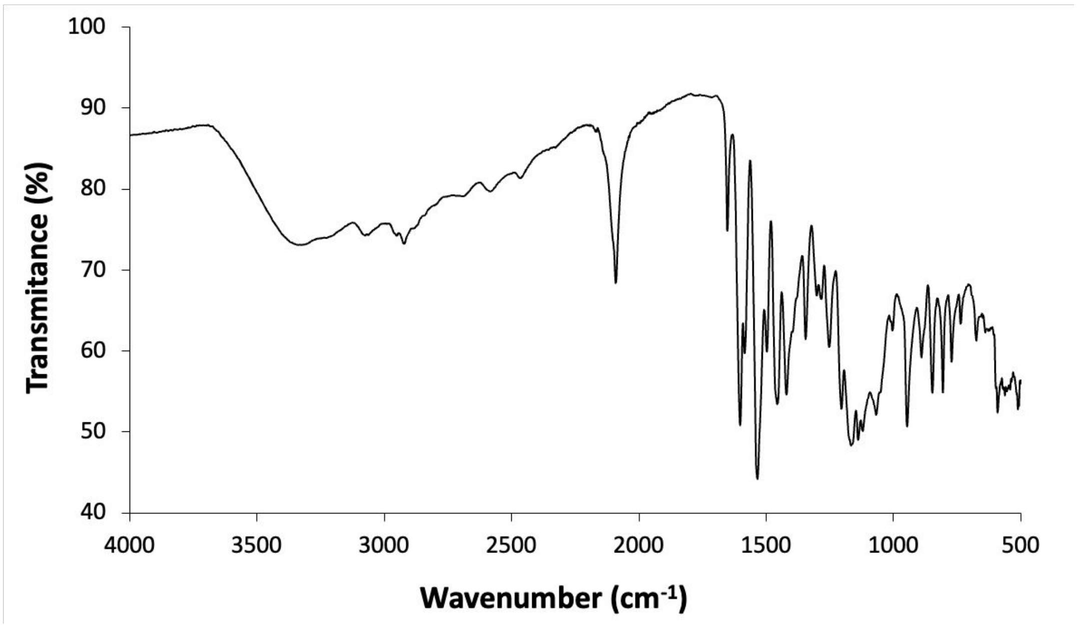

Figure S3. FTIR-ATR spectrum of Pyflav (**4**).

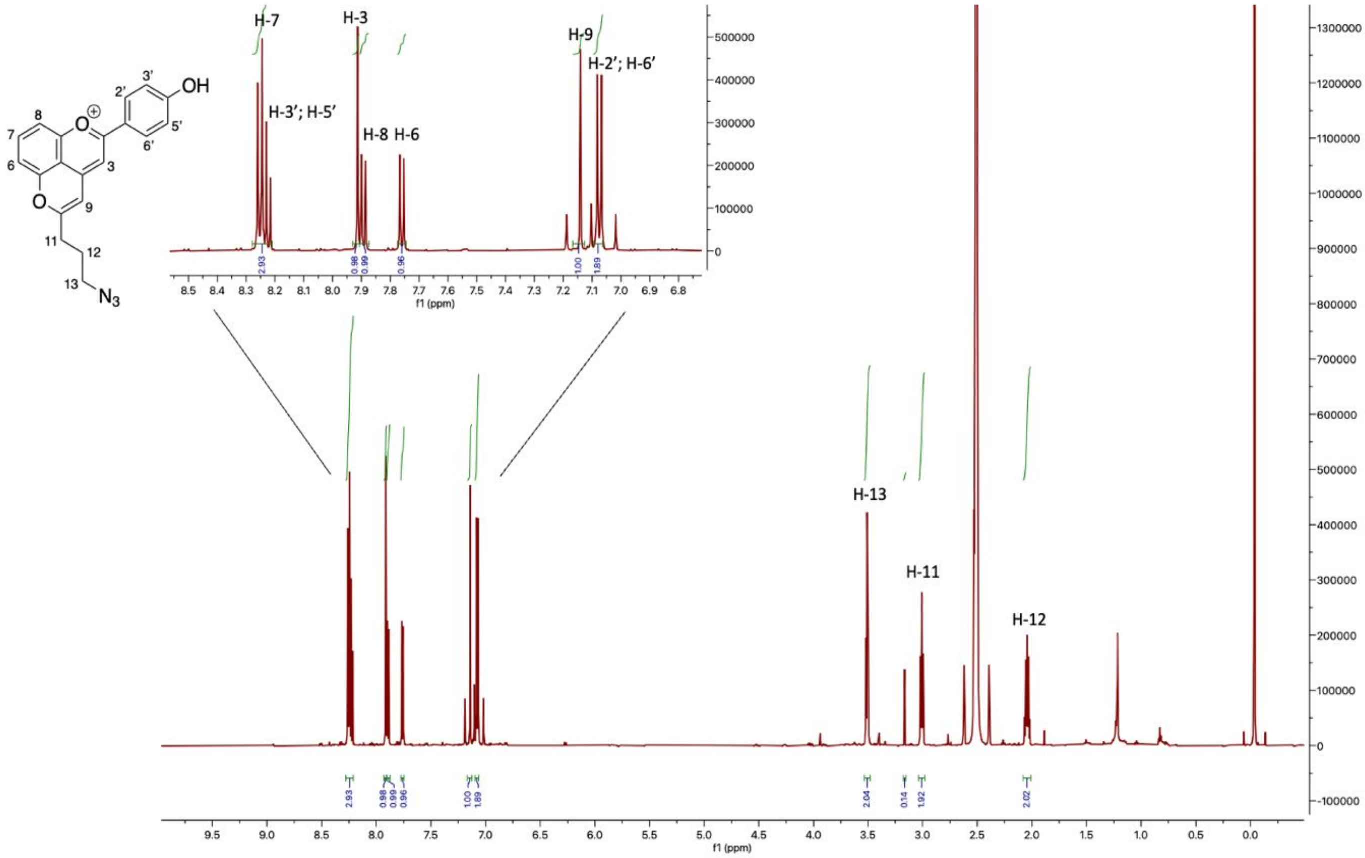

Figure S4. ^{1}H NMR (600.13 MHz) spectrum of Pyflav (**4**) in DMSO-d_6/TFA (85:15).

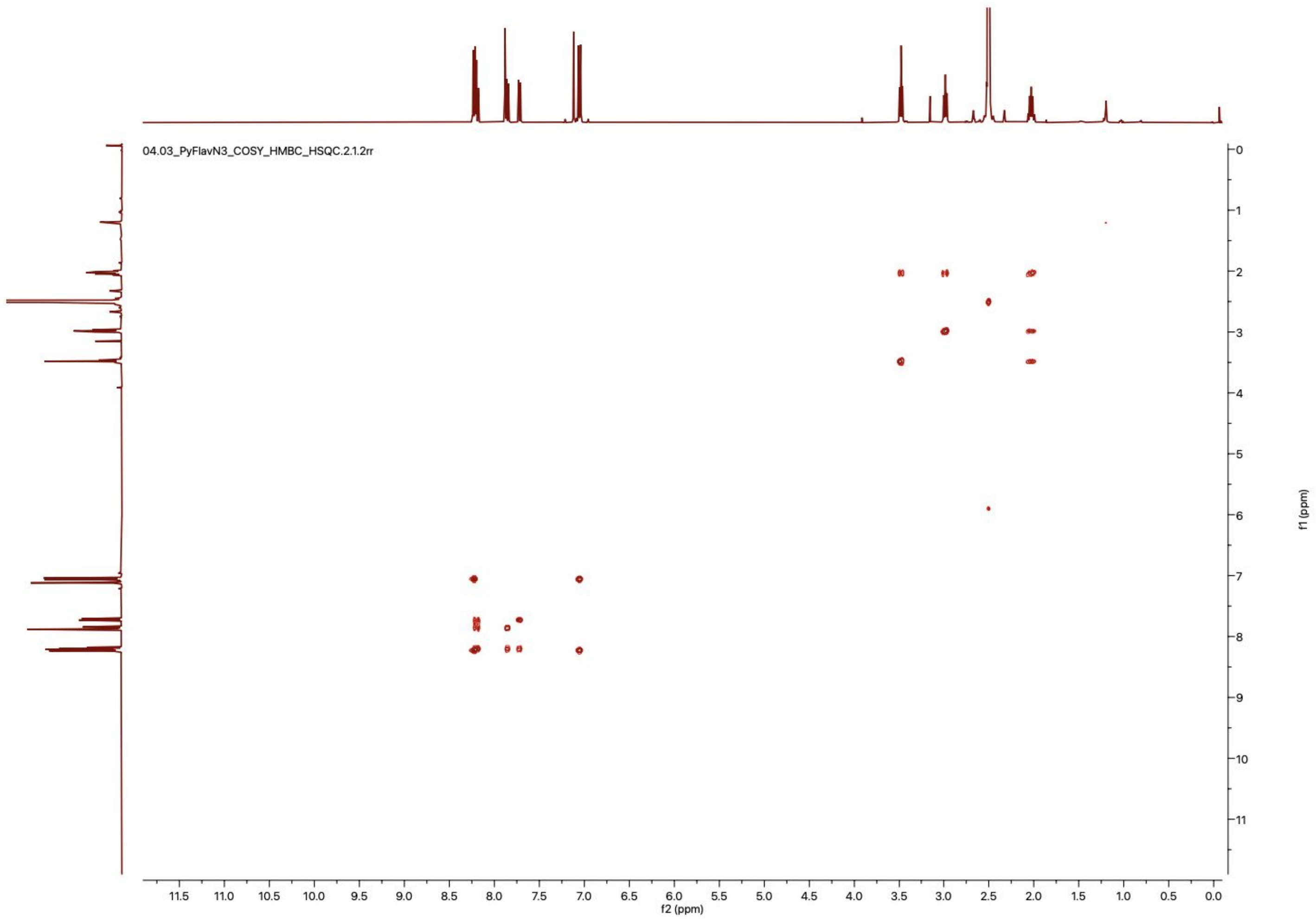

Figure S5. 2D NMR COSY spectrum of Pyflav (**4**) in DMSO-d_6/TFA (85:15).

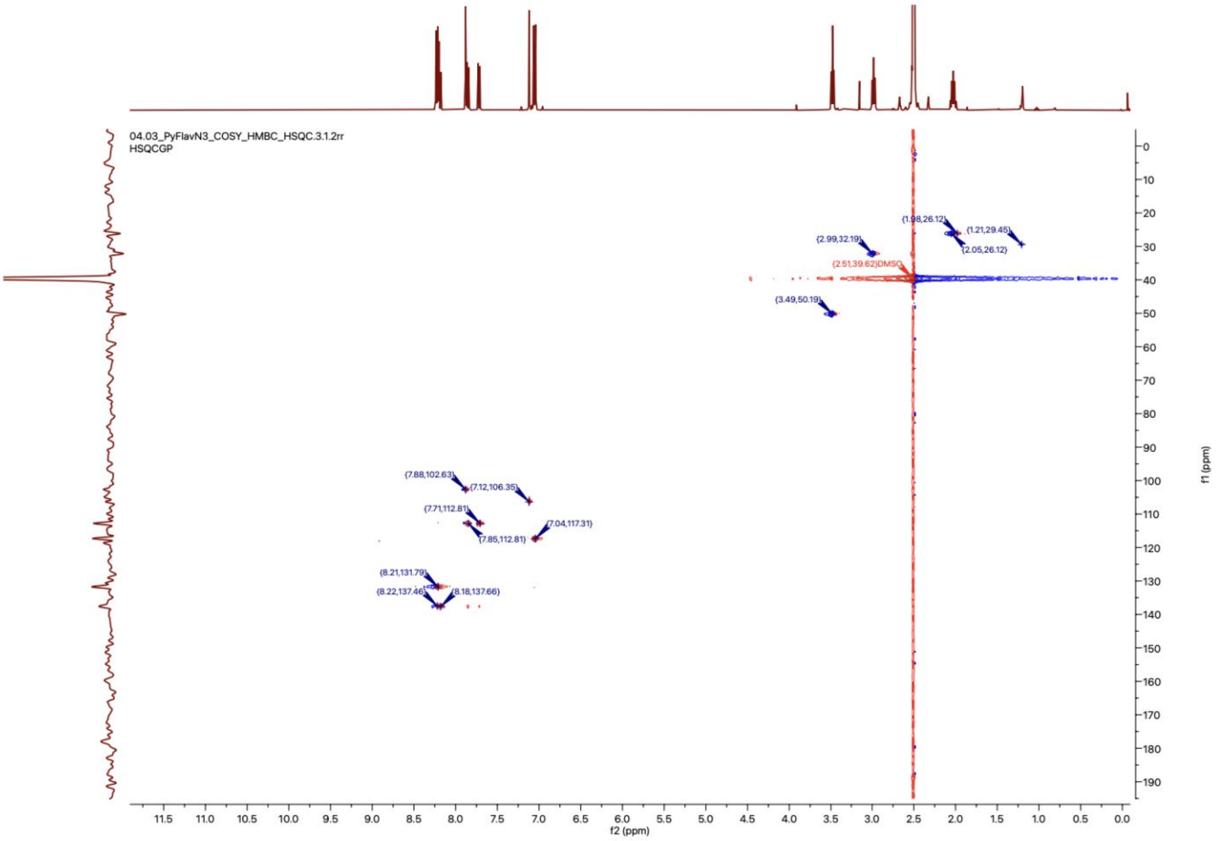

Figure S6. 2D NMR HSQC spectrum of Pyflav (**4**) in DMSO-d_6/TFA (85:15).

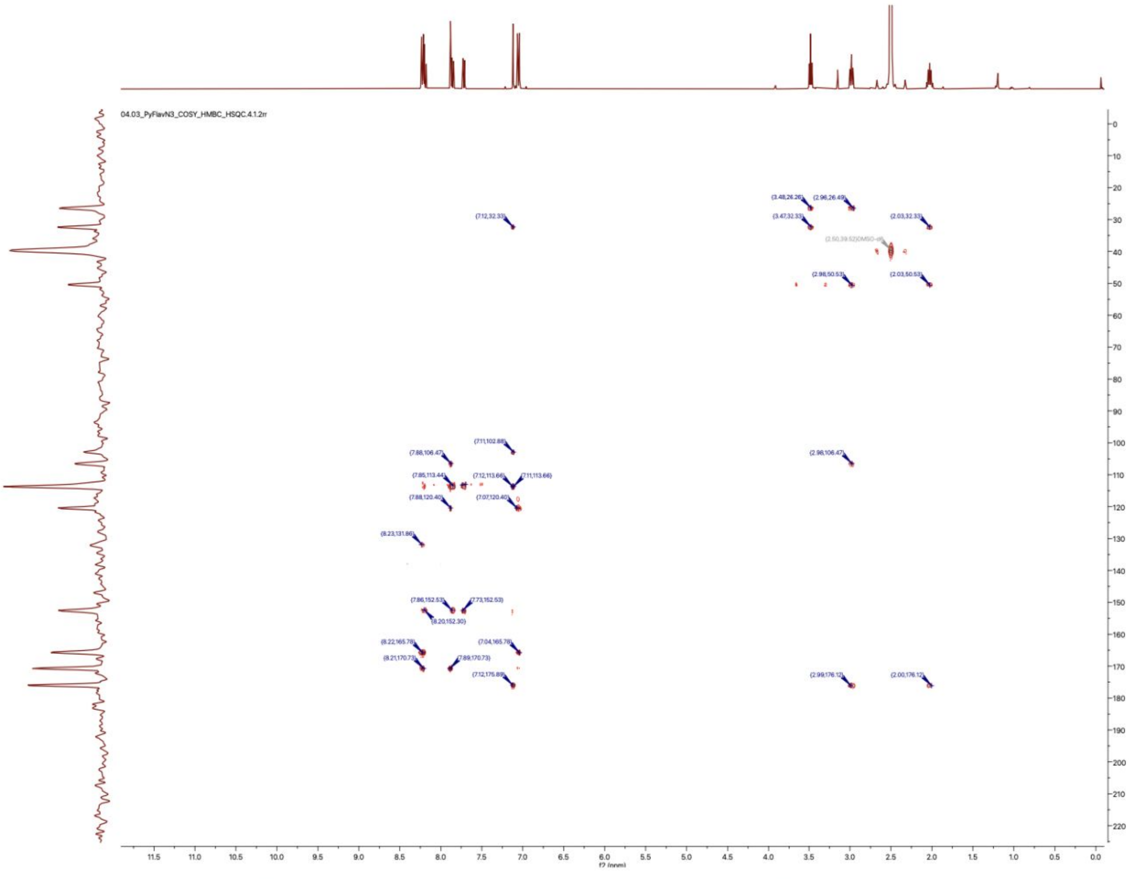

Figure S7. 2D NMR HMBC spectrum of Pyflav (**4**) in DMSO-d_6/TFA (85:15).

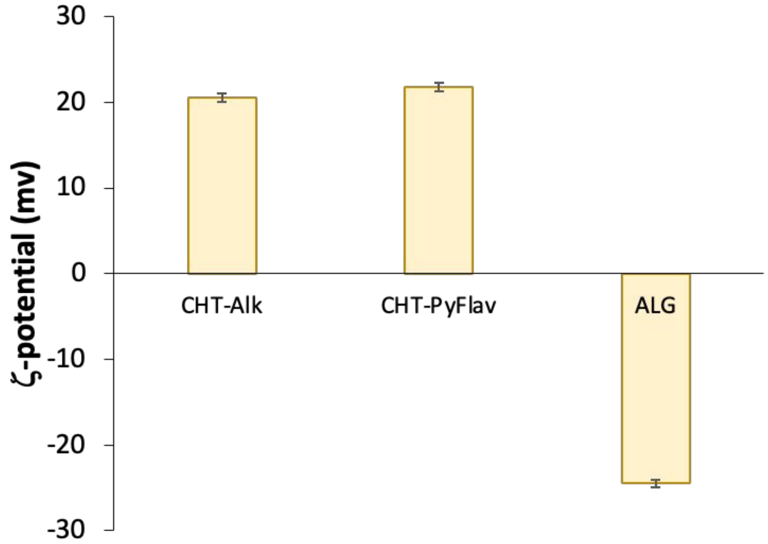

Figure S8. ξ-potential values measured for native polymers (CHT-Alk **5** and ALG) and respective conjugate (CHT-Pyflav **6**) with stock solutions 1 mg/mL in acetate buffer solutions pH 5.5 at 0.2 mg/mL.

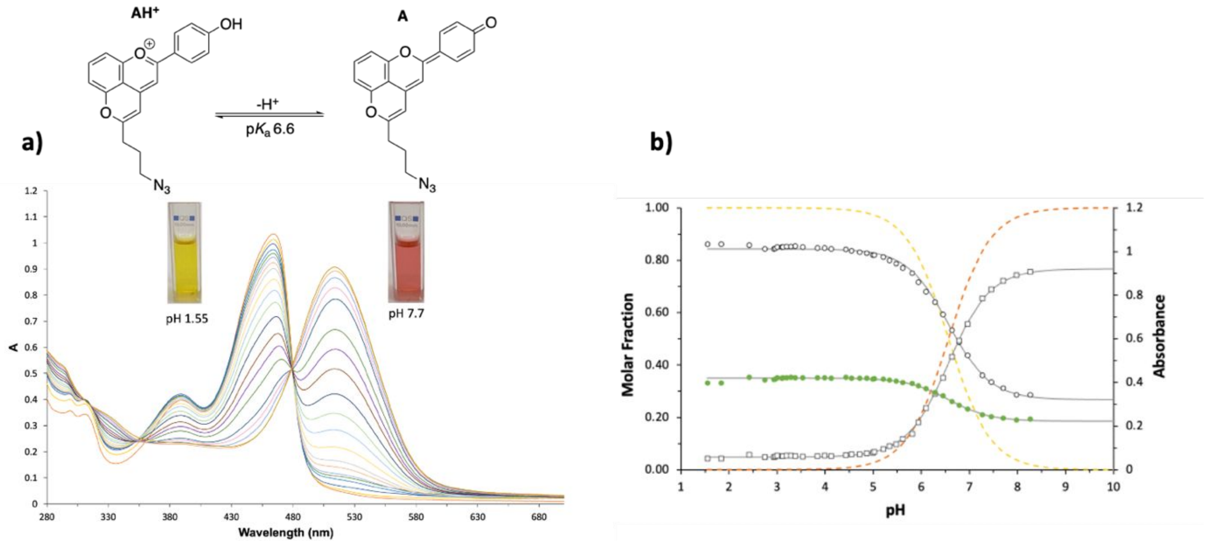

Figure S9: **a)** Spectral variations of PyFlav-N_3 **4** as a function of pH and color displayed by the equilibrium species in water/ethanol (75:25) (v/v); **b)** Molar fraction distribution and fitting of the data in function of pH to achieve pK_a = 6.6±0.5.

Table S1. Colorimetric data from the membranes in contact with buffer solutions at different pH levels.

Name	Control	pH 4	pH 5	pH 6	pH 7
Colorimeter Color Simulation					
L*	92.05	84.24	88.75	87.79	67.50
a*	3.20	1.57	3.19	1.91	10.45
b*	5.89	10.64	15.40	13.81	14.91
ΔL*	--	-7.80	-3.30	-4.25	-24.54
Δa*	--	-1.63	-0.01	-1.28	7.26
Δb*	--	4.75	9.51	7.92	9.02
ΔE*	--	9.28	10.07	9.08	27.13

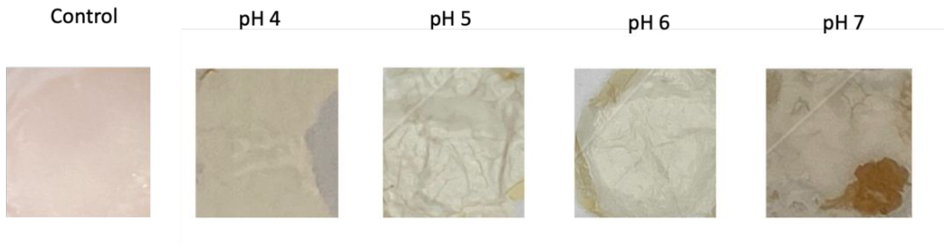

Figure S10. Images of small sections of the assembled membranes at pH values 4, 5, 6, and 7 following a 24-hour exposure in buffer solutions.

Table S2. Colorimetric data from the membranes in contact with ammonium hydroxide solutions at different concentrations.

Name	Control	NH_4OH 3.5 g·L^{-1}	NH_4OH 0.35 g·L^{-1}
Colorimeter Color Simulation			
L*	92.05	83.19	86.25
a*	3.2	6.3	4.73
b*	5.89	8.81	6.77
ΔL*	--	-8.85	-5.8
Δa*	--	3.11	1.53
Δb*	--	2.92	0.88
ΔE*	--	9.83	6.06

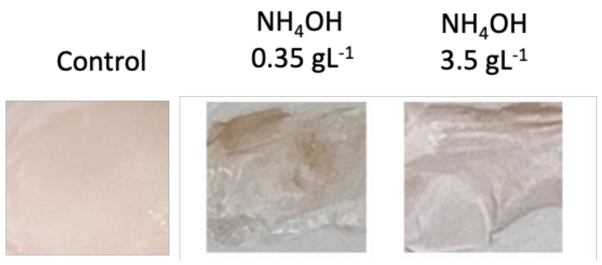

Figure S11. Images of small portions of the assembled membranes, taken after 24h of exposure in ammonium hydroxide solutions at 0.35 and 3.5 g/L.

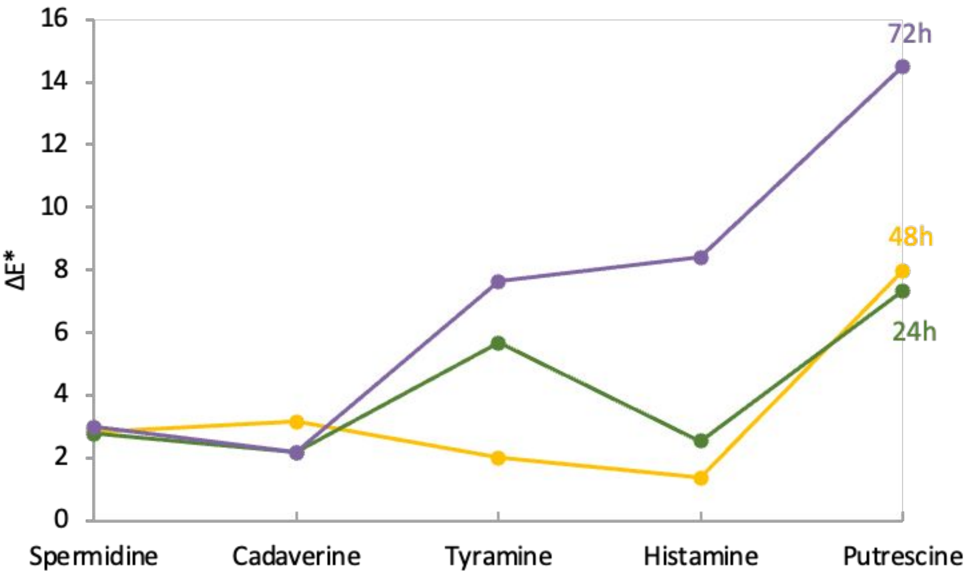

Figure S12. Chromatic variation (ΔE*) obtained for the biomembranes after exposure to biogenic amines solutions (spermidine, cadaverine, tyramine, histamine and putrescine) over time.
